# Comparative analysis of group information-guided independent component analysis and independent vector analysis for assessing brain functional network characteristics in autism spectrum disorder

**DOI:** 10.3389/fnins.2023.1252732

**Published:** 2023-10-19

**Authors:** Junlin Jing, Benjamin Klugah-Brown, Shiyu Xia, Min Sheng, Bharat B. Biswal

**Affiliations:** ^1^The Clinical Hospital of Chengdu Brain Science Institute, MOE Key Laboratory for Neuroinformation, School of Life Science and Technology, University of Electronic Science and Technology of China, Chengdu, China; ^2^Department of Biomedical Engineering, New Jersey Institute of Technology, Newark, NJ, United States

**Keywords:** autism spectrum disorder, resting-state fMRI, intersubject variability, functional network connectivity, prediction

## Abstract

**Introduction:**

Group information-guided independent component analysis (GIG-ICA) and independent vector analysis (IVA) are two methods that improve estimation of subject-specific independent components in neuroimaging studies. These methods have shown better performance than traditional group independent component analysis (GICA) with respect to intersubject variability (ISV).

**Methods:**

In this study, we compared the patterns of community structure, spatial variance, and prediction performance of GIG-ICA and IVA-GL, respectively. The dataset was obtained from the publicly available Autism Brain Imaging Data Exchange (ABIDE) database, comprising 75 healthy controls (HC) and 102 Autism Spectrum Disorder (ASD) participants. The greedy rule was used to match components from IVA-GL and GIG-ICA in order to compare the similarities between the two methods.

**Results:**

Robust correspondence was observed between the two methods the following networks: cerebellum network (CRN; |*r*| = 0.7813), default mode network (DMN; |*r*| = 0.7263), self-reference network (SRN; |*r*| = 0.7818), ventral attention network (VAN; |*r*| = 0.7574), and visual network (VSN; |*r*| = 0.7503). Additionally, the Sensorimotor Network demonstrated the highest similarity between IVA-GL and GIG-ICA (SOM: |*r*| = 0.8125). Our findings revealed a significant difference in the number of modules identified by the two methods (HC: *p* < 0.001; ASD: *p* < 0.001). GIG-ICA identified significant differences in FNC between HC and ASD compared to IVA-GL. However, in correlation analysis, IVA-GL identified a statistically negative correlation between FNC of ASD and the social total subscore of the classic Autism Diagnostic Observation Schedule (ADOS: pi = −0.26, *p* = 0.0489). Moreover, both methods demonstrated similar prediction performances on age within specific networks, as indicated by GIG-ICA-CRN (*R*^2^ = 0.91, RMSE = 3.05) and IVA-VAN (*R*^2^ = 0.87, RMSE = 3.21).

**Conclusion:**

In summary, IVA-GL demonstrated lower modularity, suggesting greater sensitivity in estimating networks with higher intersubject variability. The improved age prediction of cerebellar-attention networks underscores their importance in the developmental progression of ASD. Overall, IVA-GL may be appropriate for investigating disorders with greater variability, while GIG-ICA identifies functional networks with distinct modularity patterns.

## 1. Introduction

Autism spectrum disorder (ASD) is a neurodevelopmental disorder that affects social communication and interaction and causes restricted, repetitive behaviors and interests. It is characterized by a diverse range of symptoms and severity levels, and its underlying causes are not yet fully understood. With the advancement of neuroimaging techniques, such as Electroencephalogram (EEG) and functional magnetic resonance imaging (fMRI), researchers have been able to gain new insights into the neural mechanisms of ASD. There are several studies that have proved the effectiveness of network hierarchical structure in ASD brain function using EEG data (Wadhera and Mahmud, 2022; 2023). Studies utilizing fMRI have revealed several brain regions and networks that exhibit atypical functioning in individuals with ASD, including the social brain network, default mode network, and mirror neuron system. Additionally, researchers have explored the functional network connectivity between different brain regions and how this connectivity may be disrupted in ASD.

Numerous fMRI studies have explored the neural underpinnings of ASD and have revealed changes in brain function and connectivity in various regions, including the prefrontal cortex, amygdala, and cerebellum. For instance (Kleinhans et al., 2008) found reduced prefrontal cortex activity during a social judgment task in children with ASD, indicating that this region may be crucial to social cognition impairments in ASD. Another study (Shukla et al., 2011) observed increased amygdala activity in response to emotional faces in children with ASD, indicating altered emotional processing. A recent review (Rafiee et al., 2022) the authors summarized the latest ASD developments on tasks and resting states. For instance, Lawrence et al. (2020) found more activity in lateral frontal cortex and insula activity compared to HC, indicating that these regions may be crucial to social reward in ASD. Another study Rausch et al. (2016) showed significantly reduced connectivity in visuospatial and superior parietal areas in ASD, supporting underconnectivity theory of autism. Although fMRI has yielded valuable insights into the neural basis of ASD and may help with its diagnosis and treatment, further research is necessary to fully comprehend the underlying neural mechanisms of ASD and their role in the disorder’s development and progression.

There are a number of data-driven methods that are widely used for analyzing resting-state fMRI. Seed-based analysis is a method in which a voxel from a specific region of interest (ROI) is correlated with every voxel in the brain. This method has been used to identify functional connectivity networks in the brain (Biswal et al., 1995; Greicius et al., 2003). Machine learning is a statistical approach that can be used to learn from data and make predictions. It has been used in a growing number of ASD studies (Liu et al., 2021; Santana et al., 2022) to classify and predict brain disorders from fMRI data (Zhuang et al., 2018; Wang et al., 2022; Kunda et al., 2023).

Independent component analysis (ICA) (Mckeown et al., 1998; Damoiseaux et al., 2006) is a data driven method that has been widely used to identify and analyze brain networks. This approach of decomposing fMRI data into a linear mixture of independent components (ICs) allows researchers to identify components that are subsequently grouped as brain functional networks. Moreover, spatial ICA [sICA (Mckeown et al., 1998; Jafri et al., 2008)] has become a popular method (spatial dimension is higher than temporal dimension) accounting for spatial coherence to uncover spatially distributed data such as fMRI images and useful for identifying brain networks involved in resting-state functional connectivity. Nonetheless, temporal ICA (tICA) has also performed strongly in blind source separation (Biswal and Ulmer, 1999; Calhoun et al., 2001a; Smith et al., 2012).

Although ICA is widely used to analyze fMRI data, it has several limitations. Among these challenges is the need to modify the algorithm to account for the unique properties of fMRI data, such as high dimensionality, temporal autocorrelation, and noise properties. Model selection is also a critical factor in fMRI data analysis with ICA. Unlike other methods, the number of components to be extracted from the data is unknown *a priori* and must be estimated from the data. Various techniques can be used to determine the optimal number of components, including cross-validation, bootstrap resampling, and information criteria. Furthermore, interpretation of the results of ICA in fMRI studies requires expertise in neuroanatomy, cognitive neuroscience, and statistics. ICA can reveal complex patterns of neural activity that may be difficult to interpret without a deep understanding of brain function and organization. Finally, because fMRI studies typically involve multiple subjects, group-level analysis is required to identify common patterns of brain activity across individuals.

Group ICA (GICA) (Calhoun et al., 2001b; Svensén et al., 2002; Beckmann et al., 2009) is a variant of ICA specifically designed to identify common components in a group of subjects. The GICA is accomplished by applying the algorithm to decompose the fMRI data into independent components that are consistent across the group. In a commonly used implementation, a two-step approach is used in which each subject’s data is first decomposed into independent components before being combined into a group level. In an alternative approach, all subjects’ data is decomposed simultaneously using a single step. One of the strengths of GICA is its ability to reveal unique group-level functional networks that may not be easily visible in individual subjects. In addition, it can increase statistical power by pooling data from different subjects and reducing the effects of noise. Although GICA allows for the direct correlation of ICs between individuals in a group, it fails to capture intersubject variabilities (ISV). To address the ISV limitation of GICA, group information-guided independent component analysis (GIG-ICA) (Du and Fan, 2013; Du et al., 2016) and independent vector analysis (IVA) (Lee et al., 2008) were proposed.

IVA is a technique that maximizes both the independence between related components and the dependency between components of different subjects (Lee et al., 2008). IVA is achieved without the use of a back reconstruction phase by simultaneously calculating and optimizing ICs for each subject. This facilitates the computation of ICs at the group level and the interpretation of the relationship between ICs at the group and individual levels. Furthermore, IVA-GL (Lee et al., 2008; Anderson et al., 2012), an advanced version of IVA, can separate time-delayed and convolved signals using the Gaussian density model (IVA-G) and the Laplace density model (IVA-L) based on higher-order frequency dependencies. Previous studies (Dea et al., 2011; Michael et al., 2014) showed that IVA-GL outperformed GICA in capturing ISV in simulated fMRI data.

GIG-ICA is another technique that can obtain precise subject-specific functional networks by optimizing the independence of multiple components and improving the correspondence between each group-level IC and its associated subject-specific IC. The resulting subject-specific networks generated from the identified ICs have comparable physiological meanings, allowing for comparisons between subjects. Several studies have applied GIG-ICA to investigate functional networks in neurological diseases (Du et al., 2014, 2015, 2017; Zhi et al., 2018; Fattahi et al., 2021). These results imply that GIG-ICA can provide accurate subject-specific functional networks that are similar across participants and have physiological significance.

The advantages of GIG-ICA and IVA-GL over traditional GICA have been demonstrated in several studies (Ma et al., 2013; Michael et al., 2014; Du et al., 2016). For a comparative analysis, it was found that IVA-GL outperformed GIG-ICA in terms of identifying subject-specific signal sources as well as higher ISV though GIG-ICA detected a more stable modularity structure of FNC in healthy subjects (Du et al., 2017). Besides, IVA-GL has also been demonstrated in capturing variability, in disease subjects such as schizophrenia (Gopal et al., 2016).

In this study, we hypothesized that GIG-ICA and IVA-GL will show different brain network properties. To examine this, we compared the patterns of community structures of the FNC using features obtained from a case-control group comprising of ASD and HC. Additionally, we examined the differences in variance between the two methods to identify abnormal functional networks derived from the two groups. Finally, although network component features can be obtained from both GIG-ICA and IVA-GL to the best of our knowledge no studies have compared the prediction performance of both methods, here we used component features to predict age and WASI (three measures of IQ), respectively.

## 2. Methods and materials

This study obtained fMRI data from a publicly available Autism Brain Imaging Data Exchange (ABIDE) database (Di Martino et al., 2014). For the following analysis and prediction, we used 75 ASD patients and 102 healthy controls with ages ranging from 6.5 to 39.1 years old, scanned at New York University Langone Medical Center. ASD subjects were included based on the autism criteria in Diagnostic and Statistical Manual of Mental Disorders, 4th Edition, Text Revision (DSM-IV-TR) (American Psychiatric Association, 2000). Additionally, these subjects were acquired with a 3-T Siemens Allegra scanner. The main parameters of functional images for the resting-state are as follows: repetition time (TR)/Echo time (TE) = 2000/15 ms, flip angle = 90°, number of slices = 33, slice thickness = 4 mm, and voxel size = 3.0 × 3.0 × 4.0 mm^3^. T1-weighted images were acquired with the following parameters: TR/TE = 2530/3.25 ms, flip angle = 7°, slice thickness = 1.33 mm, and voxel size = 1.3 × 1.0 × 1.3 mm^3^. More details on the subject collection, exclusion criteria, and data parameters can be obtained from the website.^1^

### 2.1. Data preprocessing

We utilized Statistical Parametric Mapping 12 (SPM12) software^2^ and Data Processing Assistant for Resting-State fMRI^3^ to preprocess the T1-weighted images and rs-fMRI data, respectively. To preprocess the functional imaging, we took the following steps: (1) first ten time points were discarded to ensure stable magnetic resonance imaging signals at the beginning of the scan; (2) we performed head motion correction using rigid-body translation and rotation and then excluded subjects with a maximum motion greater than 3 mm or 3°; (3) trilinear interpolation with degrees of freedom was used to coregister the anatomical images with mean functional image; (4) we used the DARTEL algorithm to segment the T1-weighted image of each subject and obtained GM, WM, and CSF; (5) to reduce the residual effects of motion and other non-neuronal factors, we performed regression of the interference signal, including 24 head motion parameters, signals of WM and cerebrospinal fluid (CSF); (6) the rs-fMRI images were normalized from native space to the Montreal Neurological Institute (MNI) space with a voxel size of 3 × 3 × 3 mm^3^; (7) all normalized images were smoothed with an 8 mm full width at half maximum Gaussian kernel (FWHM).

### 2.2. Networks component estimation

We used IVA-GL and GIG-ICA to estimate components for analysis in this work. They can be available in Group ICA for fMRI toolbox (GIFT).^4^ Specifically, the main procedure steps of IVA-GL (Adali et al., 2014; Michael et al., 2014) are as follows: (1) performing subject-level PCA to each subject; (2) applying IVA-GL to estimate the SMs and TCs on each subject’s data. For GIG-ICA (Du et al., 2017), there are mainly five steps: (1) performing subject-level PCA on each subject; (2) using group-level PCA on subject-level PCA data of the temporal concatenation; (3) applying Infomax algorithm (Bell and Sejnowski, 1995) on reduced data to obtain group ICs; (4) identifying and removing artifact group components, and then computing individual components using remaining non-artifact group ICs (Du et al., 2016); (5) estimating individual TCs. The estimated components are *z*-scored on IVA-GL and GIG-ICA after completing all steps.

To obtain the statistical threshold of components of the two methods and to reduce the noise of estimation, *z*-scored *t*-test maps were computed using the MANCOVAN toolbox for ASD and HC. Next, the correspondence of components was performed using the greedy rule between IVA-GL and GIG-ICA. The paired correlation matrix were implemented and then we choose valid ICs from the matrix by checking whether the correlation value is greater than the set threshold. Finally, after excluding the mismatched networks, we selected the common resting-state networks across both methods and named them based on the Automated Anatomical Labeling (AAL) atlas (Tzourio-Mazoyer et al., 2002). The higher-order and lower-order networks were then organized for subsequent analysis.

### 2.3. Functional network connectivity and its modularity analysis

To explore the potential differences in community structure between the two techniques, we employed the Brain Connectivity Toolbox (BCT^5^) to calculate modularity measures of the FNC matrix. First, we generated subject-specific FNC matrices by calculating Pearson correlation coefficients between the time courses (TCs) of paired networks, followed by computing the mean FNC matrix across ASD subjects. To enhance the efficiency of our analysis, we applied Fisher’s *r*-to-*z* transformation to the original functional weight matrix. We utilized the Louvain algorithm, a modular community detection algorithm known for its efficiency and effectiveness (Blondel et al., 2008), to identify the hierarchical community structure and to determine the modularity *Q* value using the Girvan–Newman model (Girvan and Newman, 2002; Newman and Girvan, 2004), which reflects the quality of the community structure. A higher *Q* value represents a more stable modular structure. The modular segmentation results and corresponding *Q* value were used to analyze the FNC matrix. Finally, we performed a permutation test to calculate the group differences in module measures.

We used a two-sample *t*-test to examine the statistical difference of FNC, and the results were FDR corrected. This analysis helped determine if the two methods could capture FNC differences between ASD and HC. Additionally, we calculated the relationship between FNC and clinical measures using paired components of TCs. To consider both linear and nonlinear information, we employed a boosted method that leverages nonlinear information to enhance the linear effect (Motlaghian et al., 2022). The definition of the boosted method is as follows:


PC+sign(PC)×NMI

Where PC represents Pearson correlation and NMI represents normalized mutual information. In this case, the FNC matrix regressed the influence of gender, age and handedness scores. We have removed subjects that had abnormal or missing clinical measures. The Mahalanobis distance from the bivariate mean of the resampled data was used to identify and remove outliers from the data set (Samuel Schwarzkopf et al., 2012). Then, Shepherd’s pi correlation was applied to the data after outlier removal to measure the correlation between the two variables (Samuel Schwarzkopf et al., 2012).

### 2.4. Estimating spatial differences

Differences in the spatial statistics between ASD and HC based on two methods, IVA-GL and GIG-ICA, were assessed using three aspects. Initially, a cluster-level two-sample *t*-test was conducted on *z*-score maps to determine differences in weighted amplitude between the two groups. The results were corrected for multiple comparisons using FDR correction. Based on the significant spatial differences in pairing components between the two methods, we also displayed the histogram of amplitude values using the voxel with the most remarkable difference for each subject. This allowed us to obtain the distribution differences between the two groups. Secondly, we calculated voxelwise differences in variance maps by subtracting the variance between groups (HCs – ASDs) for each component. Finally, since the variance distribution across voxels was non-normal between subjects, a nonparametric test was utilized to identify differences in variance maps for each component between ASDs and HCs. These statistical analyses enable the identification of not only the differences in mean and variance between ASD and HC but also the IVA-GL and GIG-ICA’s characteristics in capturing variability. We also expected to discover significant differences between ASD and HC using these two methods. For instance, IVA-GL can reveal the variability of some networks, but GIG-ICA cannot, and vice versa.

### 2.5. Predictive model

**Feature selection**: to reduce the original spatial feature dimension of 54,263, we applied feature selection based on the CPM model (Shen et al., 2017), which considers the correlation between the connectivity matrix and behavioral measure. However, we used a stricter threshold value of *p* < 0.001 to determine the relationship between the original spatial networks and behavioral measures. This process resulted in obtaining a different number of voxels for each network associated with demographic variables (age and IQ), which were subsequently used for prediction.

**LASSO model**: the LASSO penalty regression is a linear model that is commonly used to estimate sparse coefficients (Friedman, 2010). This regression technique helps to prevent overfitting by applying a penalty function to compress the coefficients of variables. Mathematically, the minimized objective function of LASSO regression is:


$$\begin{array}{c} \min\\ \omega \end{array}\frac{1}{2N}{\left\|X\omega -y\right\|}_{2}^{2}+\alpha {\left\|\omega \right\|}_{1}$$

Where N is the sample size, α is a constant, and ω is the coefficient vector. The whole training and prediction process was completed based on LassoCV package of sklearn in Python. Specifically, we performed a two-layer loop, with the outer loop using a leave-one-out cross-validation (LOOCV) and the inner loop using 10-fold cross-validations. In each outer loop, the optimal α value was found through the inner loop.

We employed a permutation test to assess the efficacy of our model in predicting target values, by generating an empirical null distribution to evaluate the correlation between predicted and target values. As the two datasets were not normally distributed, we utilized Spearman’s rank correlation for the permutation test, which involved rearranging the order of two independent samples 1,000 times. To evaluate our results, we also utilized root mean squared error (RMSE), a widely used metric for assessing predictions. Notably, RMSE provided a more meaningful understanding of the actual deviation, while the fixed range of correlation values made it easier to observe. Additionally, we employed the determination coefficient *R*^2^ to reflect the regression fitting effect of the prediction model.

## 3. Results

Table 1 displays the demographic information of all subjects used in this study. There were relatively no significant age differences between the two groups (*p* = 0.311).

**Table 1:** Demographics and clinical characteristics of subjects.

	HC (*N* = 105)	ASD (*N* = 79)	*p*-value
Gender (M/F)	65/10	76/26	0.047^a^
Age (mean ± SD)	14.84 ± 7.00	15.86 ± 6.33	0.311^b^
Handedness scores (mean ± SD)	40.65 ± 51.54	65.26 ± 27.51	0.000**^b^
FIQ (mean ± SD)	107.49 ± 16.45	113.23 ± 13.09	0.011*^b^
VIQ (mean ± SD)	105.44 ± 15.91	113.09 ± 12.33	0.001*^b^
PIQ (mean ± SD)	107.16 ± 20.70	110.25 ± 13.79	0.236^b^

HC, healthy control; ASD, autism spectrum disorders; M, male; F, female; FIQ, full intelligence quotient; VIQ, verbal intelligence quotient; PIQ, performance intelligence quotient (FIQ, VIQ, and PIQ are measured by Wechsler Abbreviated Scales of Intelligence; WASI). * represents the *p* < 0.05 and ** represents the *p* < 0.001.

a Chi-square.

b Two-sample *t* test.

### 3.1. Spatial component selection and pairing

The analysis in IVA-GL and GIG-ICA used a relatively high model order of IC = 50, which has been previously demonstrated to produce reliable intrinsic component networks (ICNs) (Abou-Elseoud et al., 2010). To identify nonartifactual group components, a sorting procedure was combined with visual inspection, and fractional amplitude of low-frequency fluctuations (fALFF) and dynamic range were examined for all components. In these spectral measurements, the greater the value of the independent component representing the brain network, the lower the value of the noise component (Allen et al., 2012; Gopal et al., 2016). Finally, we only selected components with fALFF greater than 0.5 for further analysis.

Two different pairing strategies were used: (1) all components with an absolute correlation coefficient (|*r*|) greater than 0.5 were utilized for functional network connectivity (FNC) analysis and spatial differences analysis in group mean; (2) for the one-to-many case, the spatial components of IVA-GL were paired with those of GIG-ICA using high correlation coefficients, and the resulting components were used for difference analysis in variance maps of HCs and individuals with ASDs. Several networks and their corresponding correlation coefficients were obtained, including the Auditory network (AUD: |*r*| = 0.6161), Cerebellum network (CRN: |*r*| = 0.7813), Dorsal attention network (DAN: |*r*| = 0.6479), Default mode network (DMN: |*r*| = 0.7263), Motor network (MTN: |*r*| = 0.6887), Salience network (SN: |*r*| = 0.6787), Sensorimotor network (SOM: |*r*| = 0.8125), Self-reference network (SRN: |*r*| = 0.7818), Ventral attention network (VAN: |*r*| = 0.7574), and Visual network (VSN: |*r*| = 0.7503). The SOM network had the highest similarity between IVA-GL and GIG-ICA.

### 3.2. FNC and modularity analysis

**Figure 1:**
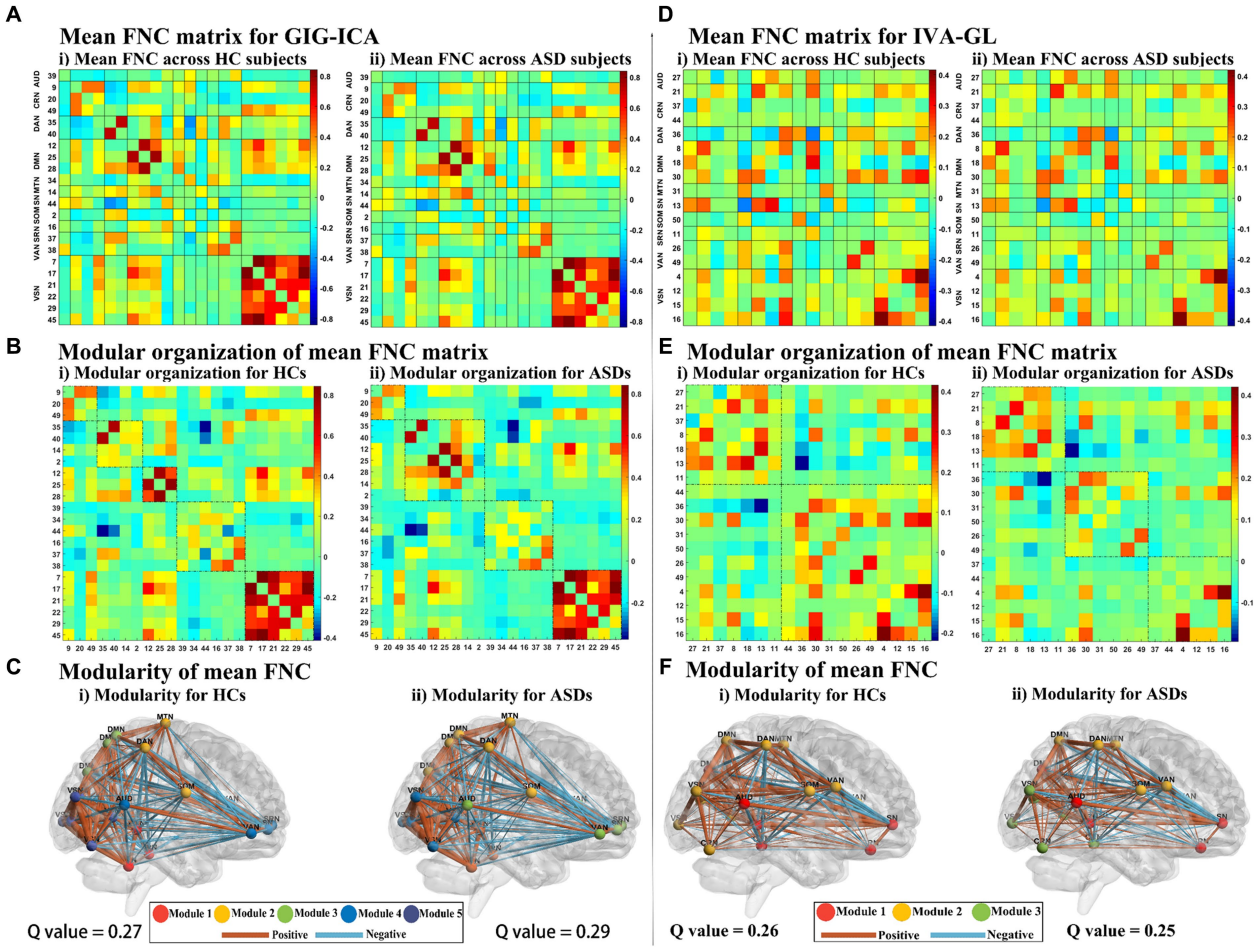
The mean FNC matrix of GIG-ICA and IVA-GL and their modularity results. **(A)** FNC correlation (averaged over ASD subjects). (i) and (ii) Show the functional network connectivity of pairing components for HC and ASD, respectively. On the left side of the FNC matrix are networks and their corresponding components, and the right side of the FNC matrix is the color bar, which represents the size of the correlation. **(B)** Modular organization of mean FNC matrix from GIG-ICA in HC and ASD. The dotted line in the matrix divides the network connectivity into multiple modules. **(C)** Modularity of the mean FNC from GIG-ICA in HC and ASD. The intrinsic networks of the modules and their functional connections were drawn in different colors, in which the thickness of the functional connections represents the connection strength. Similarly, **(D–F)** were the IVA-GL metrics corresponding to GIG-ICA.

The mean FNC matrix calculation of GIG-ICA and IVA-GL involves different numbers of matching components, which are 22 and 18, respectively. GIG-ICA exhibits stronger connection strength within the very crucial VSN network (please refer to meta-analytic database previous studies^6^), as shown in Figures 1A,D, with a maximum connection strength of 0.8, while IVA-GL only detects a maximum of 0.4. As shown in Figures 1B,E, GIG-ICA detected five and four modularities in HC and ASD, respectively. The five modules in HC identified by GIG-ICA mainly comprised of the basic function network (CRN) in module 1, low-order networks (DAN, MTN, SOM) in module 2, high-order networks (DMN) in module 3, interconnected networks (AUD, DMN, SN, SRN, VAN) in module 4 and VSN in module 5. For ASD, the four modules detected by GIG-ICA included CRN in module 1, DAN, DMN, MTN, and SOM in module 2, AUD, DMN, SN, SRN, and VAN in module 3, and VSN in module 4. On the other hand, IVA-GL detected two modularities in HC, including AUD, CRN, DMN, SN, and SRN in module 1 and CRN, DAN, DMN, MTN, SOM, VAN, and VSN in module 2. In ASD, IVA-GL detected three modularities, including AUD, CRN, DMN, SN, and SRN in module 1, DAN, DMN, MTN, SOM, and VAN in module 2, and CRN and VSN in module 3. Although GIG-ICA had a slightly higher *Q* value than IVA-GL (GIG-ICA: HC = 0.27, ASD = 0.29; IVA-GL: HC = 0.26, ASD = 0.25), the differences were small, and only provided global results.

To further explore the group differences in modules between GIG-ICA and IVA-GL, we conducted a permutation test (10,000 iterations, *p* < 0.05) for within-method and between-method comparison, as presented in Table 2. Our analysis revealed no significant differences in modularity measures between HC and ASD for both GIG-ICA and IVA-GL. Furthermore, the global modularity *Q* of GIG-ICA was not significantly different from that of IVA-GL in HC and ASD. However, we did observe significant differences in the number of modules between GIG-ICA and IVA-GL in both ASD and HC.

**Table 2:** Group differences in modularity measures for GIG-ICA and IVA-GL.

	Modularity measures	HC mean (±std)	ASD mean (±std)	*p*-value
Within-method				
GIG-ICA	Global modularity *Q*	0.28 (±0.03)	0.28 (±0.04)	0.74
	Number of modules	3.28 (±0.58)	3.30 (±0.63)	0.71
IVA-GL	Global modularity *Q*	0.27 (±0.05)	0.27 (±0.06)	0.82
	Number of modules	2.95 (±0.61)	2.84 (±0.52)	0.15

Between-method	Group	Methods		*p*-value
Global modularity *Q*	HC	GIG-ICA (0.28 (±0.03))	IVA-GL (0.27 (±0.05))	0.07
	ASD	IVA-GL (0.27 (±0.06))	GIG-ICA (0.28 (±0.04))	0.18
Number of modules	HC	GIG-ICA (3.28 (±0.58))	IVA-GL (2.95 (±0.61))	<0.001**
	ASD	IVA-GL (2.84 (±0.52))	GIG-ICA (3.30 (±0.63))	<0.001**

**Figure 2:**
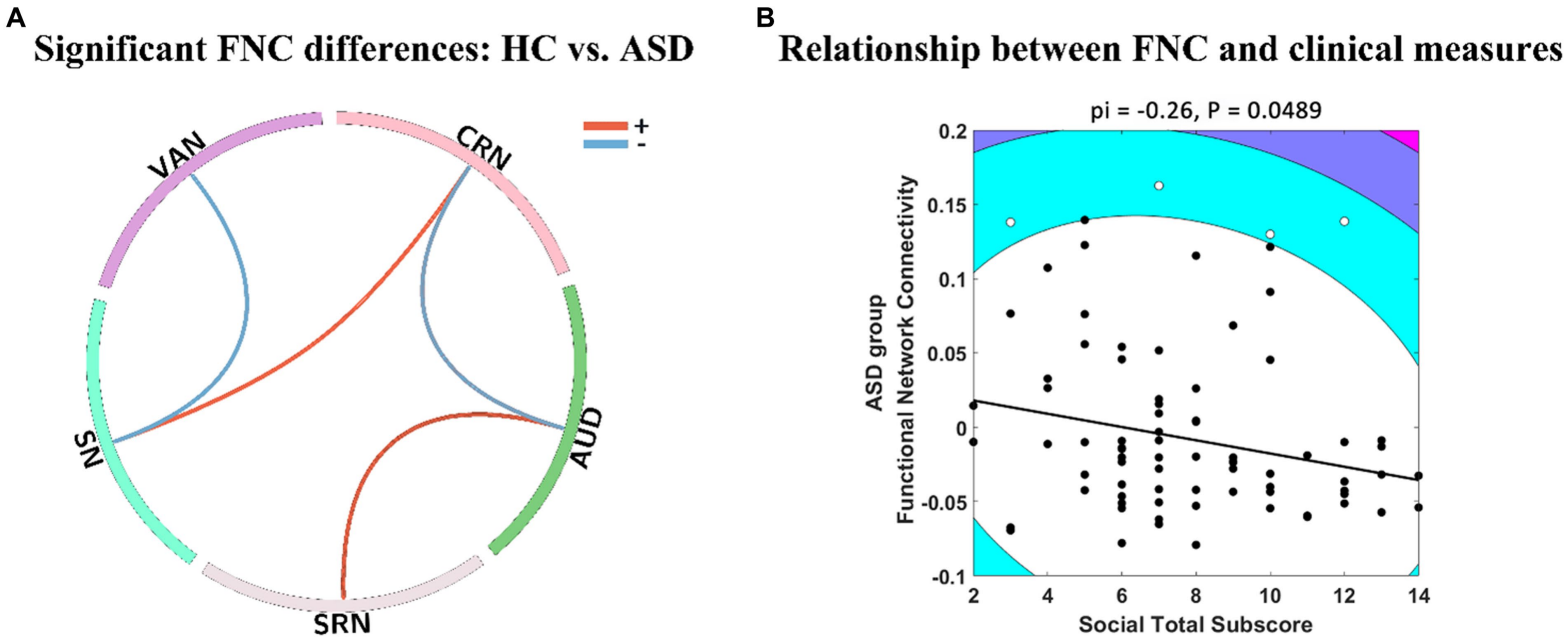
FNC analysis differences between GIG-ICA and IVA-GL. **(A)** Panel A shows significant FNC differences between HC and ASD (*p* < 0.05) using two-sample *t*-test in GIG-ICA. The line in the ring represents some significant FNC differences, where positive values indicate that HC is greater than ASD and vice versa. **(B)** Panel B illustrates negative relationship between group FNC across ASD and social total subscore of the classic ADOS in IVA-GL. The contour lines in the Shepherd’s pi correlation map indicate the bootstrapped Mahalanobis distance from the bivariate mean of the resampled data. The black points represent the data included in the correlation analysis and the white points represent the outliers excluded from Shepherd’s pi correlation analysis. The solid line represents the linear regression of the data after outlier removal and pi means Spearman correlation values after outlier removal. ADOS, autism diagnostic observation schedule.

In Figure 2A, it was observed that GIG-ICA successfully detected significant FNC effects of HC – ASD with *p* < 0.05 and FDR correction, whereas IVA did not. GIG-ICA was able to identify differences in functional connections between VAN, CRN, AUD, SRN, and SN, where SN and CRN, SRN and AUD showed positive intensity (HC > ASD). Similarly, SN and VAN, AUD, and CRN showed negative intensity (ASD > HC). On the other hand, IVA-GL demonstrated that the mean FNC values in the ASD group were negatively correlated with the social total subscore of the classic Autism Diagnostic Observation Schedule (ADOS, which serves as a standard for diagnosing ASD), as presented in Figure 2B, while GIG-ICA did not find any significant relationship (pi = 0.18, *p* = 0.2511). However, our findings demonstrated significant FNC differences between HC and ASD. The IVA-GL showed a statistically negative correlation between the FNC of ASD and the ADOS. To summarize, GIG-ICA and IVA-GL had a complementary effect on the results of FNC statistical analysis.

### 3.3. Spatial statistical analysis

**Figure 3:**
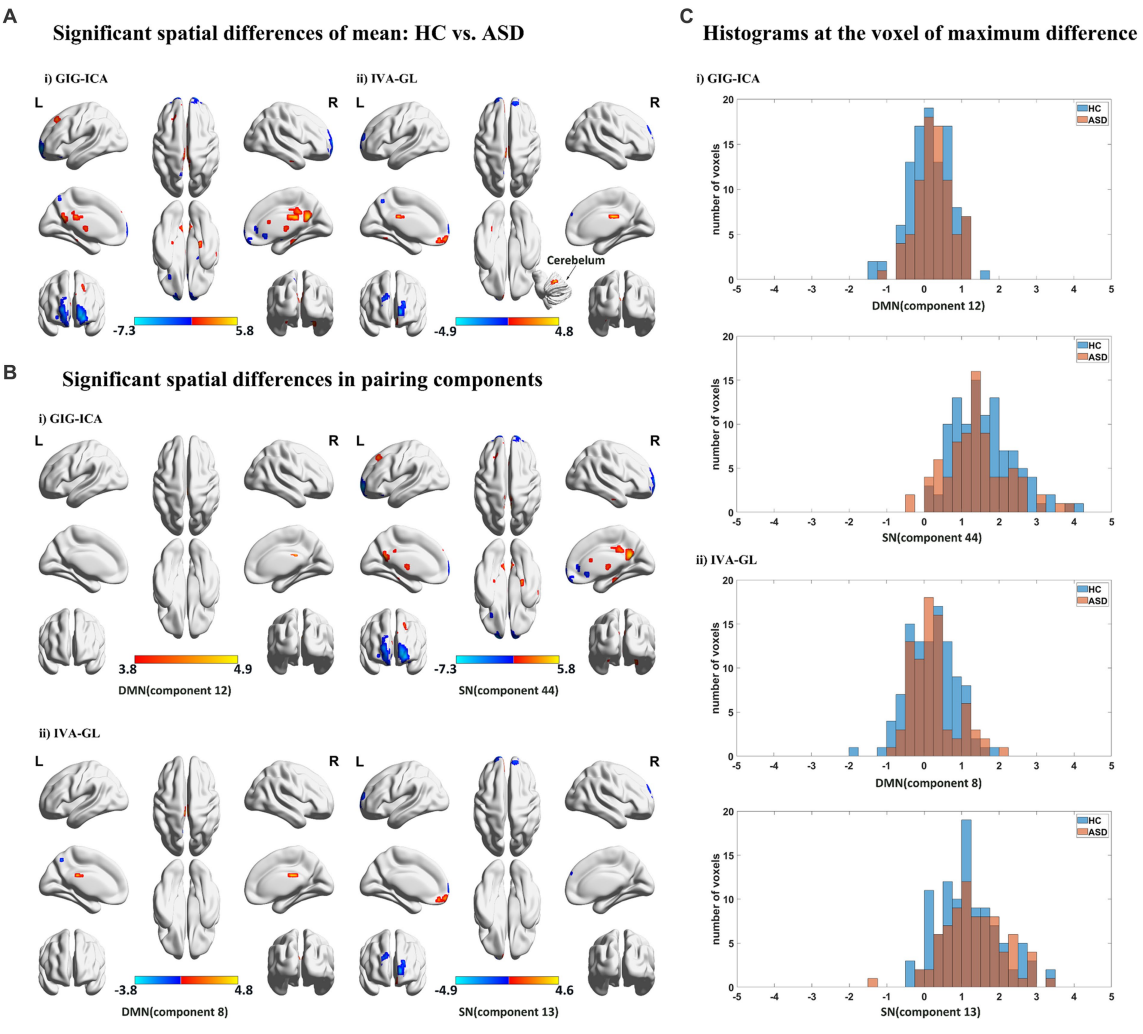
Spatial statistical analysis in mean based on GIG-ICA and IVA-GL. **(A)** Significant spatial effect (*p* < 0.05). Two sample *t*-test and FDR correction were performed and multi perspective spatial map was displayed. **(B)** Significant components of networks in HC vs. ASD (*p* < 0.05). Here are some statistically significant components, which are paired components in GIG-ICA and IVA-GL. **(C)** Histograms across subjects at cluster peak with significant difference, which come from the components with mean differences.

We utilized a two-sample *t*-test (*p* < 0.05) with FDR correction to compute spatial differences of mean, using *z*-scores *t* maps of nonartifact and paired components (Figure 3A). Results showed that GIG-ICA had significantly higher amplitudes and larger cluster size than IVA-GL, including CRN, DMN, SN, and VSN, respectively. Although GIG-ICA detected more activated areas, IVA-GL was better at identifying more important brain regions, such as the cerebellum. Significant components were matched between GIG-ICA and IVA-GL (DMN: *r* = 0.6586, SN: *r* = 0.6787) in Figure 3B. IVA-GL revealed both positive and negative DMN regions, while GIG-ICA only showed positive regions with a slight fluctuation range. On the other hand, GIG-ICA displayed a more prominent activation intensity in SN, different from IVA-GL. A histogram in Figure 3C showed the differences in weighted amplitudes of four components between HC and ASD of GIG-ICA and IVA-GL on the network, respectively, which included DMN and SN. Most voxels across subjects on SN had amplitude values between 1 and 2, while those of DMN was between 0 and 1, indicating that SN had more activities than DMN. The difference in DMN in Figure 3C was mainly evident in the distribution of ASD, while SN was concentrated in HC. These results provide significant insights into the neuroanatomy of autism.

Table 3 revealed that GIG-ICA detected more voxels compared to IVA-GL. The two-sample *t*-test *p*-values for each component at the voxel of maximum difference are as follows: SN of GIG-ICA: *t*-test *p*-value =$\;6.79\times 10^{-12}$ (HC < ASD); SN of IVA-GL: *t*-test *p*-value =$\;1.65\times 10^{-06}$ (HC < ASD); DMN of GIG-ICA: *t*-test *p*-value =$\;2.36\times 10^{-06}$ (HC > ASD); DMN of IVA-GL: *t*-test *p*-value =$\;2.83\times 10^{-06}$ (HC > ASD). The number of voxels showed significant changes after uncorrected and FDR correction, especially in DMN, with at least one cluster surviving correction. Given this situation, the uncorrected results were also presented in Table 3.

**Table 3:** Two-sample *t*-test for voxelwise group differences in the mean, based on matching components between GIG-ICA and IVA-GL.

Method	Matching components	Peak Foci/*T*-value	*p*-values (Unc)	NOV	*p*-values (Cor.)	NOV
GIG-ICA	44 (SN)	(−18, 57, −3)/−7.3418	0.015	872	0.006	592
	12 (DMN)	(3, −30, 24)/4.8750	0.019	251	0.000	23
IVA-GL	13 (SN)	(−18, 57, 3)/−4.9548	0.016	328	0.002	124
	8 (DMN)	(3, −21, 24)/4.8343	0.017	202	0.000	12

Unc, uncorrected; Cor, FDR-corrected; NOV, number of voxels.

**Figure 4:**
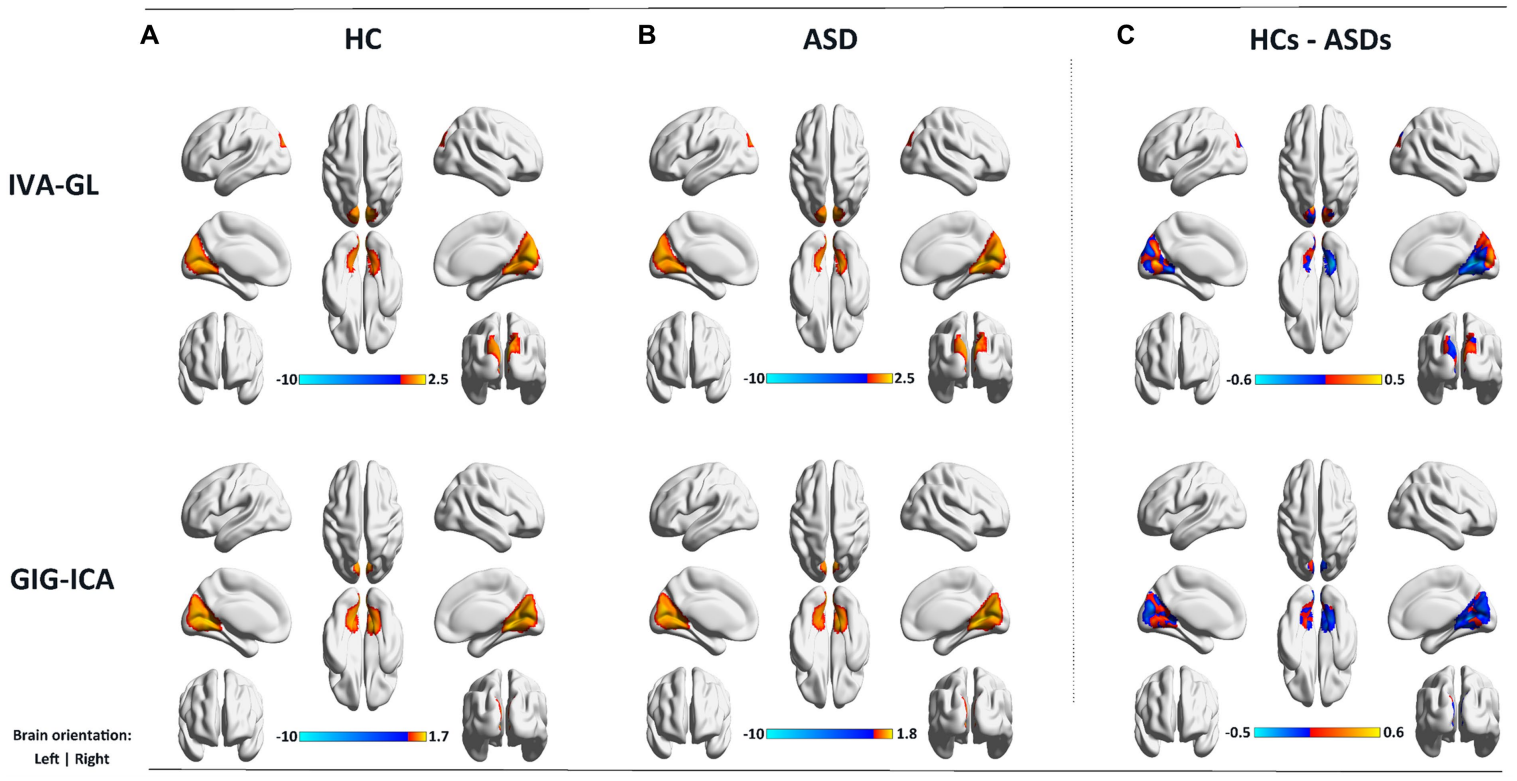
Variance maps for controls **(A)** and patients **(B)** as well as the difference **(C)** in the variance maps (HCs – ASDs) for visual network (VSN) for both IVA-GL and GIG-ICA.

In addition, using the mean gray matter across subjects (Figure 4), a corresponding mask was created to calculate the spatial difference of variance maps based on the visual network. As anticipated, IVA-GL captured more variability than GIG-ICA, and ASD exhibited higher variance than HC, a noteworthy phenomenon. The findings suggest that autism is associated with a more intricate brain network activity than control.

**Figure 5:**
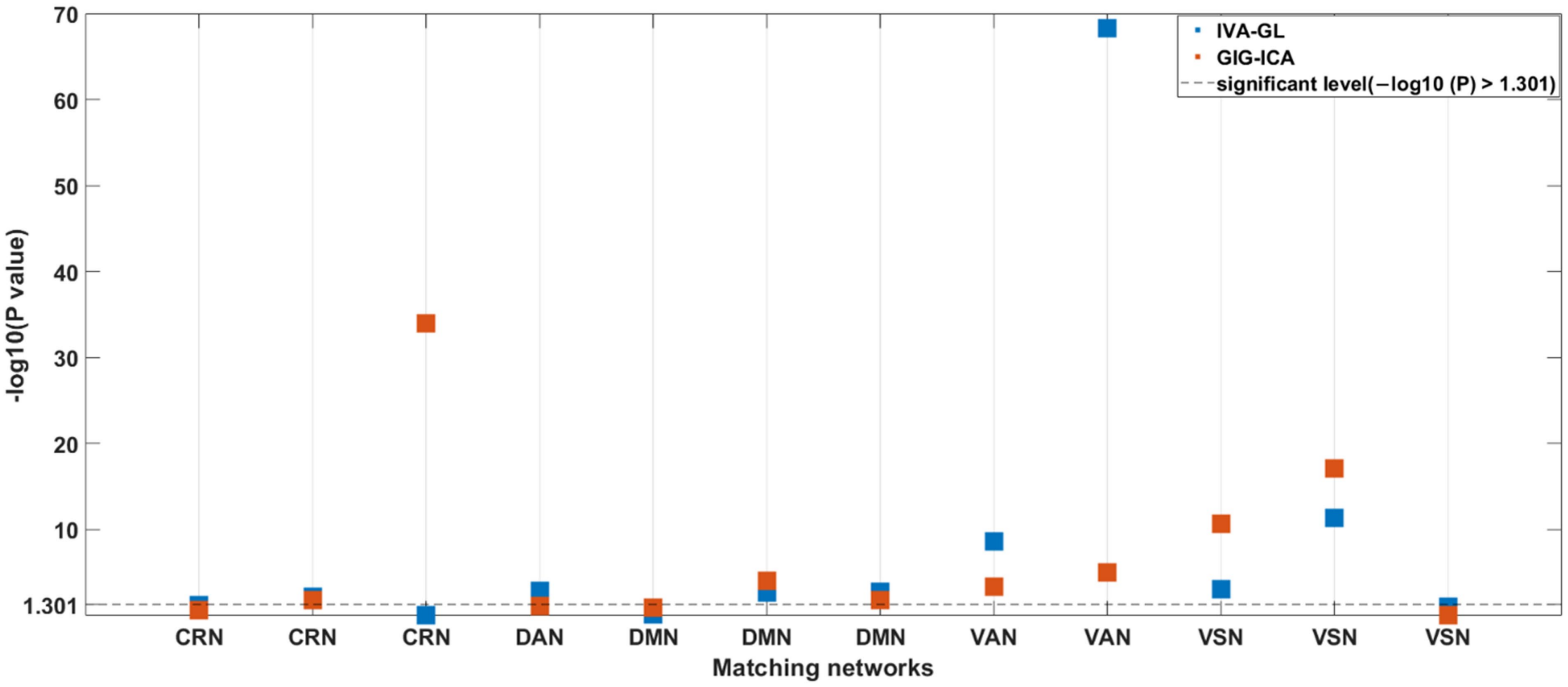
*p*-values for nonparametric test for difference in variance maps of HCs and ASDs for all matching components between two methods.

A nonparametric test for variance differences between individuals with ASD and HC was conducted, examining the mean variance maps for all pairing components. In Figure 5, negative logarithm values of *p* and significant levels were shown, with blue and red dots representing IVA-GL and GIG-ICA, respectively. The third (CRN) and fourth (DAN) columns differed particularly between the two methods, especially in CRN. The majority of networks showed significant differences (ASD > HC), except for DMN (GIG-ICA: IC = 34, *p*-value = 0.000; IVA-GL: IC = 18, *p*-value = 0.002) and VSN (GIG-ICA: IC = 22, *p*-value = 0.000; IVA-GL: IC = 12, *p*-value < 0.001). Overall, these results indicate that individuals with autism exhibit significantly higher variance than those without in CRN, DMN, VAN, and VSN.

### 3.4. Comparison of prediction for IVA-GL and GIG-ICA

**Figure 6:**
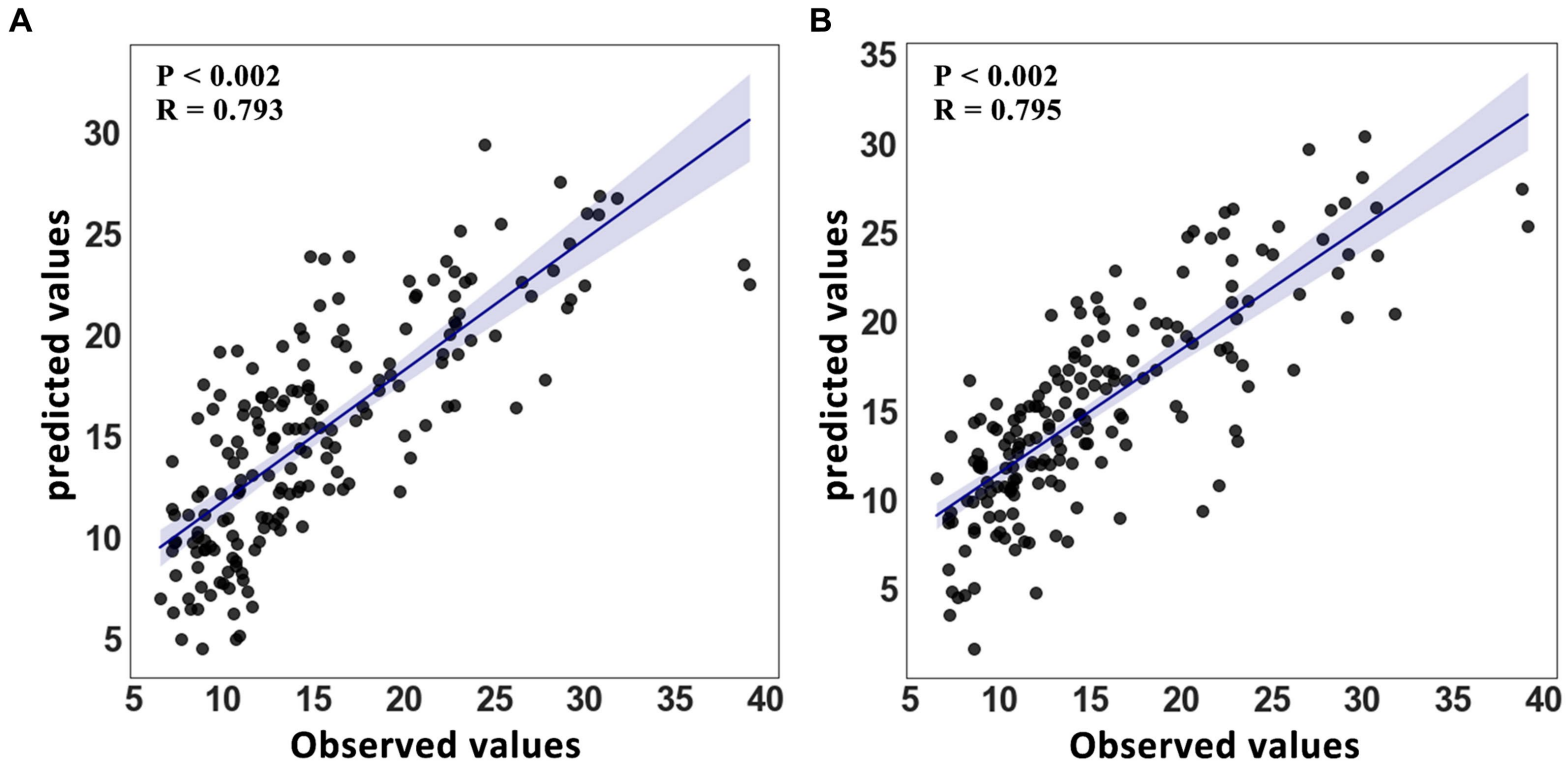
Regression model for age with 95% confidence interval. **(A)** Linear scatter plot between predicted value and observed value in VAN of IVA-GL. **(B)** A graph similar to IVA-GL for GIG-ICA’s CRN.

**Figure 7:**
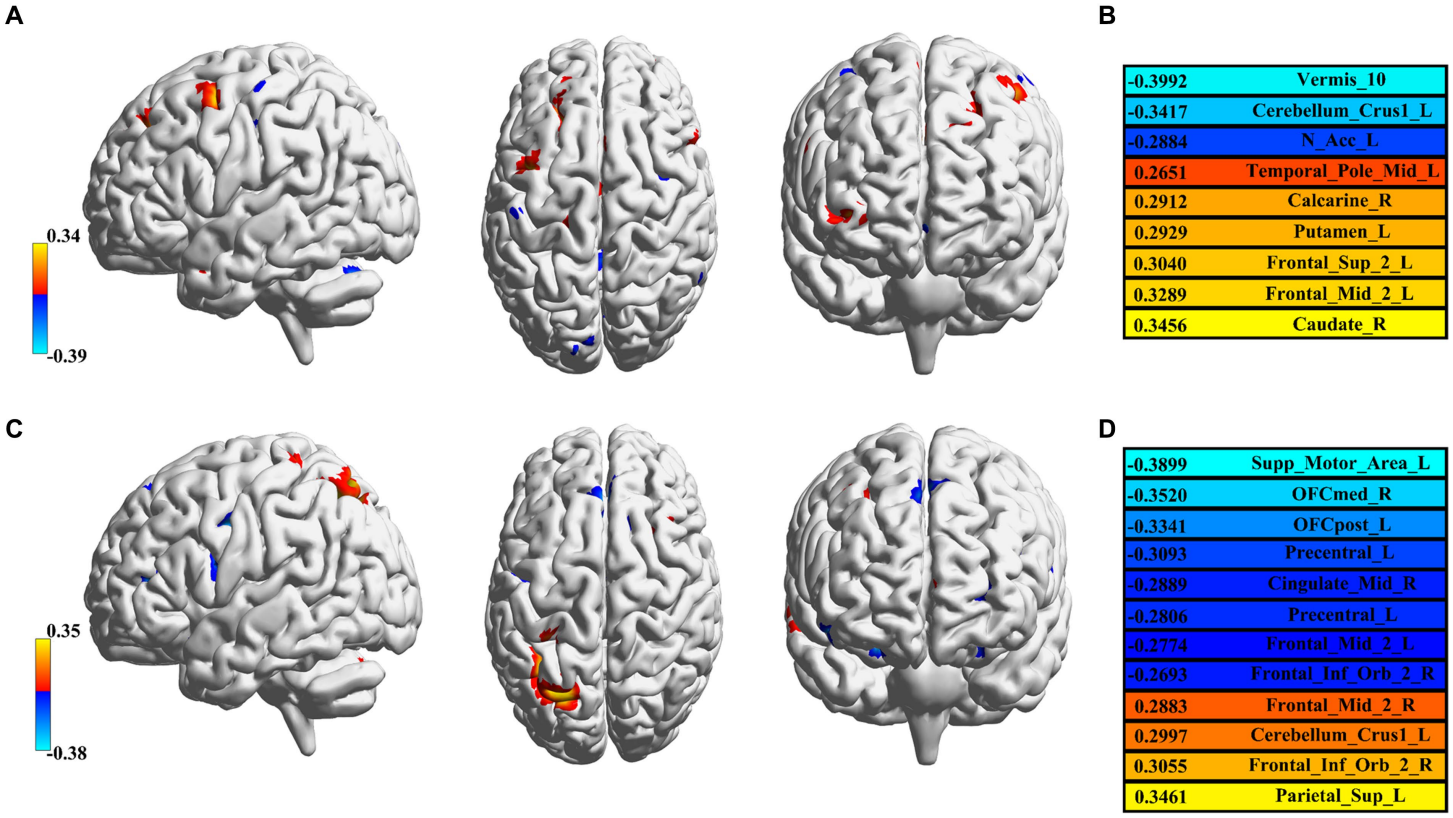
Feature visualization in GIG-ICA and IVA-GL. **(A)** CRN in GIG-ICA and its corresponding brain regions. **(B)** The list of detailed brain regions for GIG-ICA. **(C)** Corresponding brain regions for VAN in IVA-GL. **(D)** The list of detailed brain regions for IVA-GL. All brain regions are labeled according to the AAL atlas with each color in C and D corresponding to correlation values.

The feature selection process was used to determine the number of voxels for each spatial network. The feature sizes for both methods’ best-performing networks for predicting age were listed (GIG-ICA: CRN = 471; IVA-GL: VAN = 622); additional networks can be found in the Supplementary material. In terms of the determination coefficient *R*^2^ in Table 4, the GIG-ICA method had better model fitting performance compared to IVA-GL. Permutation tests were used to calculate the correlation coefficient and *p*-values to evaluate the prediction results, and a regression plot between the predicted and observed values was shown in Figure 6. The root-mean-square error (RMSE) values for IVA-GL and GIG-ICA were also displayed in Table 4, with the smallest RMSE for IVA-GL in VAN and GIG-ICA in CRN. Notably, our results indicated which network is most suitable for predicting age using these two methods. Additionally, a paired *t*-test was conducted to detect any differences in prediction values between IVA-GL and GIG-ICA, but the results showed no significant difference (*t* = −0.09, *p*-value = 0.92). The performance of all networks can be found in the Supplementary Table S1. To aid visualization, we presented brain maps in Figure 7 that displayed regions within the CRN and VAN that provided the most effective features for prediction.

**Table 4:** Comparison of GIG-ICA and IVA-GL in prediction performance.

Method	Network	*R* ^2^	RMSE
GIG-ICA	CRN	0.91	3.05
IVA-GL	VAN	0.87	3.21

However, compared to age, the three IQ measures might not be reliable predictors. For FIQ and VIQ, GIG-ICA outperforms IVA-GL (FIQ (RMSE: GIG-ICA-DMN = 7.22; IVA-CRN = 8.44); VIQ (RMSE: GIG-ICA-DMN = 7.66; IVA-VSN = 7.70)). However, for PIQ, IVA-GL surpasses GIG-ICA (RMSE: GIG-ICA-SOM = 8.99; IVA-VSN = 8.71). Additional details on these results are provided in Supplementary Table S1.

## 4. Discussion

This study investigated the potential differences between GIG-ICA and IVA-GL using resting-state data in ASD and HC. The study mainly focused on FNC measures (i.e., global modularity), spatial network differences (i.e., variance maps), and prediction performance (i.e., RMSE). We found that GIG-ICA and IVA-GL have different strengths when analyzing brain networks in HC and ASD. GIG-ICA can detect more regions and higher amplitudes, while IVA-GL is better at identifying abnormal brain regions in ASD. Both methods are equally effective at calculating the difference in variance maps between HC and ASD and demonstrated a complementary relationship in FNC analysis. Although GIG-ICA has better predictive performance, there was no significant statistical difference between the two methods.

### 4.1. FNC strengths and global modularity *Q*

The FNC strengths and global modularity *Q* in GIG-ICA were greater than those of IVA-GL. These findings align with a previous study (Du et al., 2017) that reported similar metrics in the HC dataset. However, we conducted additional statistical analyses to determine significant differences in the number of modules and global modularity *Q* within and between the two methods. We found no significant difference in the number of modules or global modularity *Q* between HC and ASD for within-method comparison. This contrasts with previous study in children (5–10 years) with ASD (Sigar et al., 2023), which found a significant difference. Given the wider age range (7.1–39.1 years) in our study, we speculate that there may be differences in the number of modules and global modularity *Q* between adult and child groups.

Regarding the between-method, we found significant differences in the number of modules between GIG-ICA and IVA-GL, regardless of HC or ASD. Given that GIG-ICA identifies more modules than IVA-GL, this result suggests that brain functional system obtained by GIG-ICA has higher integration and IVA-GL has higher segregation and result from that GIG-ICA can obtain more reliable networks compared to IVA-GL (Du et al., 2017). Additionally, it may also reflect that GIG-ICA has higher randomness of functional brain network (Rudie et al., 2013; Keown et al., 2017; Henry et al., 2018; Sigar et al., 2023). Our study further indicates no significant difference in global modularity *Q* between GIG-ICA and IVA-GL. Notably, GIG-ICA detected differences in FNC between HC and ASD, suggesting that it preserved the group relationship and identified potential differences. Similarly, IVA-GL identified a statistically negative correlation between the FNC of ASD and the ADOS, indicating its capability to extract ASD variability.

### 4.2. Spatial network differences

To investigate spatial variations in variance and mean, we employed various statistical measures to pair GIG-ICA and IVA-GL components. Although IVA-GL was previously used to analyze schizophrenia (Gopal et al., 2016), we assessed and compared the ability of the two methods to detect spatial network differences in ASD. During the pairing between the two methods, SN and DMN were found to be prominent, and we observed that GIG-ICA showed larger network areas and higher *t*-value differences, while IVA-GL identified cerebellar regions important to ASD. Our finding aligned with a prior investigation conducted by Du and colleagues, demonstrating that GIG-ICA is useful for evaluating coherent networks across subjects, whereas IVA-GL can estimate subject-specific networks (Du et al., 2017). Interestingly, both methods demonstrated similar amplitudes at the voxels of maximum difference. In terms of spatial variance analysis, IVA-GL detected more variability than GIG-ICA, consistent with previous studies comparing it with other methods (Lee et al., 2008; Michael et al., 2014; Laney et al., 2015). Moreover, we found that the group difference pattern (ASD > HC; ASD < HC) obtained from GIG-ICA was almost identical to IVA-GL, but there were differences in the statistical discrimination of some networks (CRN (GIG-ICA: IC = 49, *p*-value < 0.001; IVA-GL: IC = 44, *p*-value = 0.838), DAN (GIG-ICA: IC = 40, *p*-value = 0.074; IVA-GL: IC = 36, *p*-value = 0.001)), indicating that the two methods had comparable abilities to detect variance map differences.

### 4.3. Prediction performance

Using a regression model, GIG-ICA and IVA-GL were compared to determine which data-driven method was best for predicting age. Unlike typical feature selection methods in machine learning, we employed a statistical correlation method to identify the voxels most relevant to age and eliminated the effects of gender and handedness. To comprehensively evaluate the prediction results, we utilized a permutation test and the confidence coefficient *R*^2^. The findings indicated no significant difference between the two methods in predicting age. Nevertheless, we displayed the predictive features of two networks in Figure 7. In GIG-ICA’s CRN, the vermis and caudate was found to have the strongest negative and positive correlation with age, respectively. Similarly, for IVA-GL, the motor area correlated negatively with age, while the parietal was positively correlated. Additionally, the prediction accuracy for IQ is not as high as that for age, possibly because of the greater variance in its data.

### 4.4. Limitations

Despite the thorough evaluation of GIG-ICA and IVA-GL performance using various metrics, our study does have some limitations. Firstly, our subjects comprise different age groups, which might require an additional analysis to examine their differences. Since our study mainly focused on methodological differences, we did not investigate this aspect. Therefore, further studies will be required. Secondly, although a boosted method was used to calculate the FNC matrix, further research is needed to verify the effect of extracting network information. Considering that the *p*-value in Figure 2B is close to the statistically significant boundary, more work may be required to check its validity. Lastly, full IQ was determined by averaging the available performance and verbal IQ scores for each diagnostic group. Given the significant differences in FIQ and VIQ between the HC and ASD groups, the predictive characteristics might vary between them. While our comparison of prediction performance primarily focused on both methods of the two groups as a whole, further research may be necessary to delve deeper into the predictive characteristics of each group individually.

## 5. Conclusion

In conclusion, this study revealed that GIG-ICA and IVA-GL have distinct capabilities in identifying brain network modules in HC and ASD, with a complementary effect on FNC statistical analysis. GIG-ICA can detect more regions with higher amplitudes in spatial network differences, while IVA-GL can identify more networks associated with ASD. In addition, both methods can accurately determine the difference in variance maps between HC and ASD, with GIG-ICA having a better predictive performance (GIG-ICA-CRN (*R*^2^ = 0.91, RMSE = 3.05, *R* = 0.795) and IVA-VAN (*R*^2^ = 0.87, RMSE = 3.21, *R* = 0.793)), although not significantly different from IVA-GL (*t* = −0.09, *p*-value = 0.92). Finally, the study offers further insights into using different data-driven methods to examine neurological disorders of resting-state fMRI.

## Data availability statement

Publicly available datasets were analyzed in this study. This data can be found at: http://fcon_1000.projects.nitrc.org/indi/abide/abide_I.html.

## Author contributions

JJ, SX, and MS: organized and preprocessed the data. JJ and BK-B: formal analysis, statistical analysis and results, and writing the first draft. BK-B and BB: revision and editing. All authors contributed to the article and approved the submitted version.

## Footnotes

^1^ http://fcon_1000.projects.nitrc.org/indi/abide/abide_I.html

^2^ http://www.fil.ion.ucl.ac.uk/spm/software/spm12

^3^ http://rfmri.org/DPARSF

^4^ https://trendscenter.org/software/

^5^ https://sites.google.com/site/bctnet/

^6^ https://neuroquery.org/
