# Supplementary material for: Comparative analysis of group information-guided independent component analysis and independent vector analysis for assessing brain functional network characteristics in autism spectrum disorder
Source: Front Neurosci. 2023 Oct 19;17:1252732. doi: 10.3389/fnins.2023.1252732 (PMC10620743; doi:10.3389/fnins.2023.1252732)
Supplement: Supplementary file 1 [file Data_Sheet_1.docx]

Supplementary Material

**Comparative Analysis of Group Information-Guided Independent Component Analysis (GIG-ICA) and Independent Vector Analysis (IVA-GL) for Assessing Brain functional Network Characteristics in Autism Spectrum Disorder (ASD)**

Junlin Jing^1^, Benjamin Klugah-Brown^1*^, Shiyu Xia^1^, Ming Sheng^1^, Bharat B. Biswal^1,2*^

*** Correspondence:** Bharat B. Biswal: [bbiswal@gmail.com](mailto:bbiswal@gmail.com) or Benjamin Klugah-Brown: bklugah@gmail.com

# Supplementary Tables

**Supplementary Table 1.** Comparison of GIG-ICA and IVA-GL in prediction performance for all paired networks.

|  |  |  | **AGE** | | | | **FIQ** | | | | **VIQ** | | | | **PIQ** | | | |
| --- | --- | --- | --- | --- | --- | --- | --- | --- | --- | --- | --- | --- | --- | --- | --- | --- | --- | --- |
|  | **Netwo-rk** | **IC** | **Feat-ure size** | **R^2^** | **RM-SE** | **R** | **Feat-ure size** | **R^2^** | **RM-SE** | **R** | **Feat-ure size** | **R^2^** | **RM-SE** | **R** | **Feat-ure size** | **R^2^** | **RM-SE** | **R** |
| **GIG-ICA** | **AUD** | 39 | 694 | 0.88 | 3.81 | 0.69 | 47 | 0.58 | 9.00 | 0.64 | 73 | 0.57 | 9.49 | 0.59 | 53 | 0.52 | 9.96 | 0.55 |
|  | **CRN** | 9 | 411 | 0.91 | 3.33 | 0.77 | 91 | 0.68 | 9.00 | 0.65 | 97 | 0.64 | 8.59 | 0.68 | 62 | 0.63 | 8.80 | 0.67 |
|  |  | 20 | 817 | 0.91 | 3.54 | 0.71 | 14 | 0.37 | 10.02 | 0.53 | 22 | 0.45 | 9.52 | 0.59 | 37 | 0.58 | 9.32 | 0.64 |
|  |  | 49 | 471 | 0.92 | 3.05 | 0.79 | 49 | 0.55 | 9.13 | 0.63 | 58 | 0.52 | 9.14 | 0.61 | 71 | 0.55 | 9.56 | 0.60 |
|  | **DAN** | 35 | 574 | 0.90 | 3.29 | 0.75 | 79 | 0.56 | 10.07 | 0.54 | 44 | 0.56 | 8.92 | 0.60 | 72 | 0.58 | 9.93 | 0.53 |
|  |  | 40 | 409 | 0.90 | 3.26 | 0.74 | 76 | 0.61 | 9.21 | 0.65 | 49 | 0.54 | 9.00 | 0.60 | 45 | 0.60 | 9.08 | 0.67 |
|  | **DMN** | 12 | 550 | 0.91 | 3.79 | 0.68 | 137 | 0.84 | 7.22 | 0.80 | 115 | 0.74 | 7.66 | 0.71 | 95 | 0.69 | 9.48 | 0.64 |
|  |  | 34 | 573 | 0.84 | 3.19 | 0.79 | 160 | 0.67 | 8.92 | 0.67 | 110 | 0.73 | 8.30 | 0.72 | 95 | 0.58 | 9.42 | 0.63 |
|  |  | 25 | 601 | 0.90 | 3.27 | 0.76 | 96 | 0.65 | 9.24 | 0.64 | 54 | 0.54 | 9.48 | 0.59 | 96 | 0.66 | 9.56 | 0.58 |
|  |  | 28 | 295 | 0.91 | 3.20 | 0.79 | 36 | 0.55 | 9.35 | 0.63 | 18 | 0.35 | 10.10 | 0.48 | 74 | 0.65 | 9.03 | 0.69 |
|  | **MTN** | 14 | 513 | 0.91 | 3.27 | 0.77 | 49 | 0.65 | 8.24 | 0.69 | 103 | 0.61 | 9.01 | 0.59 | 36 | 0.47 | 10.35 | 0.54 |
|  | **SN** | 44 | 584 | 0.89 | 3.06 | 0.79 | 105 | 0.61 | 10.15 | 0.58 | 99 | 0.73 | 8.00 | 0.73 | 64 | 0.55 | 10.34 | 0.54 |
|  | **SOM** | 2 | 1280 | 0.93 | 3.34 | 0.76 | 107 | 0.65 | 9.31 | 0.66 | 72 | 0.55 | 9.57 | 0.56 | 85 | 0.62 | 9.00 | 0.58 |
|  | **SRN** | 16 | 1286 | 0.94 | 3.54 | 0.72 | 76 | 0.54 | 9.69 | 0.55 | 50 | 0.50 | 9.58 | 0.58 | 59 | 0.60 | 9.23 | 0.57 |
|  | **VAN** | 37 | 844 | 0.92 | 3.50 | 0.72 | 57 | 0.68 | 8.44 | 0.72 | 64 | 0.63 | 8.86 | 0.65 | 52 | 0.64 | 9.10 | 0.70 |
|  |  | 38 | 752 | 0.87 | 3.34 | 0.74 | 75 | 0.60 | 9.39 | 0.59 | 84 | 0.65 | 8.70 | 0.65 | 63 | 0.56 | 10.02 | 0.56 |
|  | **VSN** | 7 | 270 | 0.91 | 3.14 | 0.76 | 95 | 0.57 | 9.76 | 0.60 | 79 | 0.45 | 9.60 | 0.52 | 76 | 0.60 | 9.60 | 0.63 |
|  |  | 17 | 300 | 0.84 | 3.18 | 0.73 | 67 | 0.62 | 9.35 | 0.61 | 74 | 0.65 | 8.69 | 0.66 | 82 | 0.65 | 9.26 | 0.66 |
|  |  | 45 | 517 | 0.88 | 3.37 | 0.76 | 60 | 0.67 | 8.45 | 0.70 | 30 | 0.55 | 8.95 | 0.65 | 54 | 0.60 | 9.08 | 0.65 |
|  |  | 22 | 353 | 0.92 | 3.42 | 0.70 | 95 | 0.61 | 9.30 | 0.63 | 37 | 0.44 | 9.61 | 0.58 | 95 | 0.62 | 9.18 | 0.60 |
|  |  | 21 | 410 | 0.92 | 3.37 | 0.73 | 35 | 0.59 | 9.15 | 0.65 | 43 | 0.54 | 9.09 | 0.63 | 52 | 0.58 | 9.90 | 0.59 |
|  |  | 29 | 610 | 0.86 | 3.74 | 0.70 | 60 | 0.62 | 9.44 | 0.62 | 59 | 0.64 | 8.45 | 0.65 | 69 | 0.60 | 9.40 | 0.61 |
| **IVA-GL** | **AUD** | 27 | 260 | 0.68 | 3.83 | 0.62 | 67 | 0.61 | 8.95 | 0.65 | 133 | 0.57 | 9.31 | 0.60 | 55 | 0.59 | 9.55 | 0.62 |
|  | **CRN** | 21 | 457 | 0.83 | 3.57 | 0.71 | 44 | 0.57 | 9.13 | 0.64 | 49 | 0.60 | 8.89 | 0.65 | 50 | 0.50 | 9.98 | 0.57 |
|  |  | 37 | 331 | 0.78 | 3.82 | 0.73 | 101 | 0.69 | 8.44 | 0.70 | 87 | 0.61 | 8.95 | 0.64 | 73 | 0.61 | 9.87 | 0.61 |
|  |  | 44 | 221 | 0.59 | 4.36 | 0.56 | 18 | 0.58 | 10.78 | 0.43 | 19 | 0.32 | 10.22 | 0.42 | 17 | 0.20 | 110.03 | 0.38 |
|  | **DAN** | 36 | 535 | 0.85 | 3.89 | 0.64 | 53 | 0.53 | 9.59 | 0.57 | 45 | 0.50 | 9.43 | 0.57 | 54 | 0.57 | 9.39 | 0.63 |
|  | **DMN** | 8 | 576 | 0.87 | 3.36 | 0.75 | 103 | 0.68 | 8.58 | 0.58 | 81 | 0.59 | 8.93 | 0.63 | 91 | 0.58 | 9.77 | 0.61 |
|  |  | 18 | 362 | 0.83 | 3.30 | 0.73 | 52 | 0.43 | 10.25 | 0.49 | 69 | 0.47 | 9.92 | 0.54 | 63 | 0.57 | 9.82 | 0.60 |
|  |  | 30 | 350 | 0.83 | 3.37 | 0.73 | 38 | 0.45 | 10.05 | 0.51 | 24 | 0.44 | 9.46 | 0.60 | 57 | 0.61 | 9.19 | 0.64 |
|  | **MTN** | 31 | 350 | 0.83 | 3.44 | 0.69 | 52 | 0.52 | 9.76 | 0.58 | 96 | 0.60 | 9.04 | 0.62 | 25 | 0.33 | 11.06 | 0.43 |
|  | **SN** | 13 | 612 | 0.80 | 3.94 | 0.66 | 86 | 0.56 | 9.33 | 0.60 | 80 | 0.65 | 8.80 | 0.64 | 68 | 0.46 | 9.99 | 0.54 |
|  | **SOM** | 50 | 1561 | 0.88 | 3.59 | 0.74 | 87 | 0.60 | 9.06 | 0.63 | 66 | 0.54 | 9.75 | 0.56 | 91 | 0.51 | 9.91 | 0.56 |
|  | **SRN** | 11 | 616 | 0.72 | 4.34 | 0.56 | 7 | 0.22 | 10.95 | 0.37 | 19 | 0.39 | 10.49 | 0.46 | 17 | 0.26 | 11.26 | 0.36 |
|  | **VAN** | 26 | 622 | 0.87 | 3.21 | 0.79 | 92 | 0.62 | 9.03 | 0.64 | 40 | 0.51 | 9.80 | 0.56 | 87 | 0.63 | 8.94 | 0.66 |
|  |  | 49 | 218 | 0.79 | 3.43 | 0.72 | 92 | 0.42 | 10.38 | 0.51 | 80 | 0.52 | 9.16 | 0.61 | 59 | 0.48 | 10.44 | 0.52 |
|  | **VSN** | 4 | 174 | 0.69 | 4.00 | 0.58 | 181 | 0.72 | 8.83 | 0.65 | 178 | 0.77 | 7.70 | 0.76 | 81 | 0.70 | 8.71 | 0.66 |
|  |  | 12 | 75 | 0.54 | 4.01 | 0.59 | 106 | 0.58 | 8.92 | 0.62 | 87 | 0.46 | 9.84 | 0.49 | 57 | 0.58 | 9.25 | 0.64 |
|  |  | 15 | 448 | 0.85 | 3.63 | 0.71 | 19 | 0.40 | 10.11 | 0.54 | 30 | 0.46 | 9.65 | 0.60 | 37 | 0.42 | 10.48 | 0.54 |
|  |  | 16 | 430 | 0.84 | 3.58 | 0.66 | 57 | 0.53 | 9.37 | 0.60 | 75 | 0.59 | 8.72 | 0.66 | 54 | 0.54 | 9.80 | 0.60 |

# Supplementary Figures


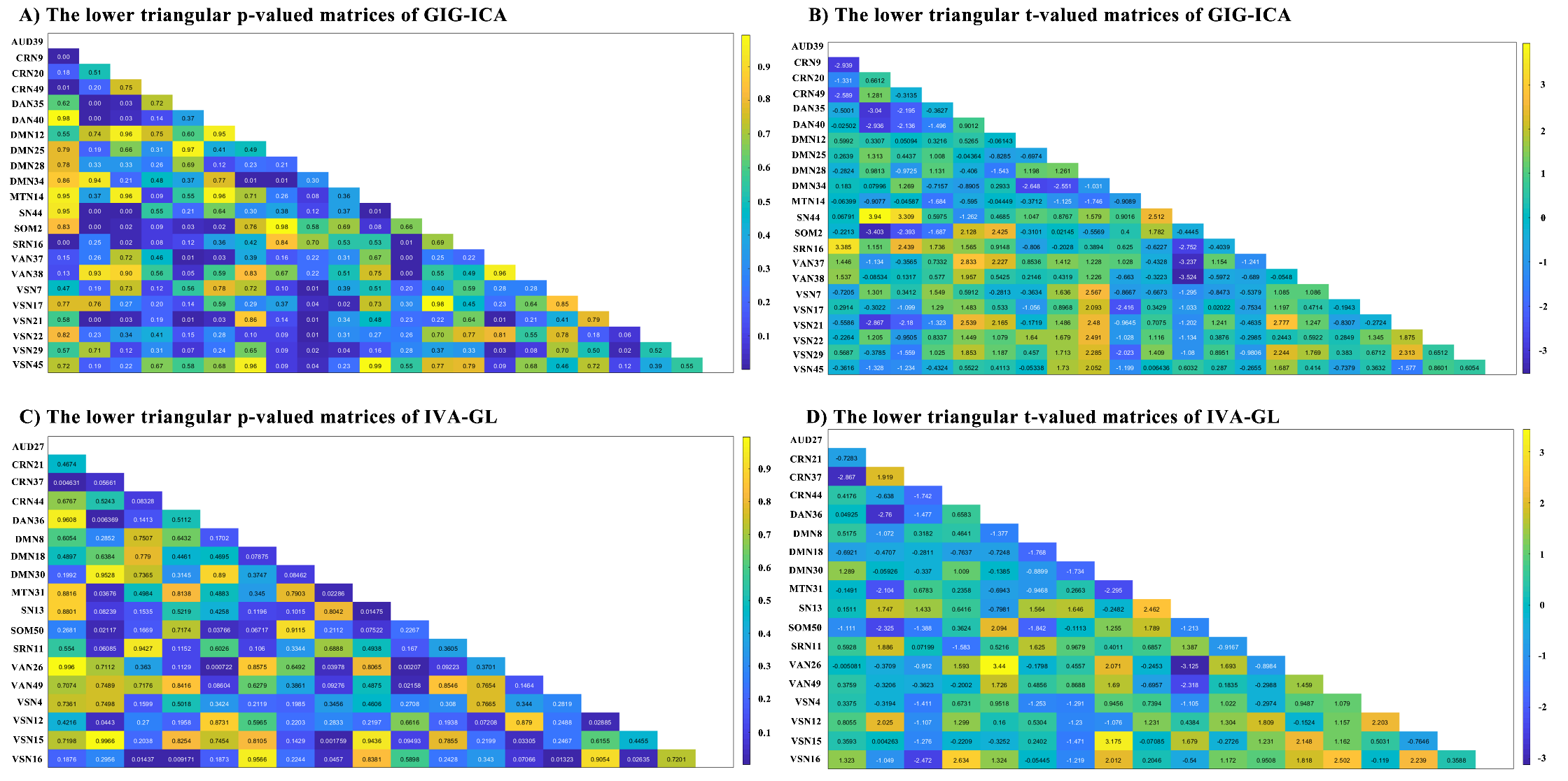


**Supplementary Figure 1.** The statistical FNC differences of HC – ASD with P < 0.05 in GIG-ICA and IVA-GL. **(A, C)** The lower triangular p-valued matrices of GIG-ICA and IVA-GL respectively. **(B, D)** The lower triangular t-valued matrices of GIG-ICA and IVA-GL respectively.

Given Figure 2A in the paper, in which GIG-ICA has significant FNC differences between HC and ASD by FDR correlation, including SN-VAN (t= -3.3805, p = 9.87e-04), SN-CRN (t= 3.6245, p = 6.22e-04), AUD-CRN (t= -2.939, p = 37.14e-04) and AUD-SRN (t= 3.385, p = 8.72e-04).
